# Supplementary material for: Intraoperative intravenous low-dose esketamine improves quality of early recovery after laparoscopic radical resection of colorectal cancer: A prospective, randomized controlled trial
Source: PLoS One. 2023 Jun 2;18(6):e0286590. doi: 10.1371/journal.pone.0286590 (PMC10237502; doi:10.1371/journal.pone.0286590)
Supplement: S2 File — (DOCX) [file pone.0286590.s002.docx]

**项目计划书**

项目名称： 小剂量艾司氯胺酮对腹腔镜结直肠癌根治术患者术后早期恢复质量的影响

申 请 人：艾艳秋

科 室：麻醉科

电 话：13607690334

联 系 人：许颖

电 话：13523550017

1. **立项依据**（研究意义、国内外研究现状及发展动态分析，需结合科学研究发展趋势来论述科学意义；或结合国民经济和社会发展中迫切需要解决的关键科技问题来论述其应用前景。附主要参考文献目录）。

结直肠癌（colorectal cancer，CRC）是临床常见的恶性肿瘤，随着生活习惯及饮食结构的改变，近年来该病发生率持续升高^[1]^。目前临床上主要以手术切除为主的综合方式进行治疗，腹腔镜结直肠癌根治术较传统开腹手术微创、并发症少，已成为临床常见术式，并且在一定程度上加快了术后恢复^[2]^，这与加速康复外科（enhanced recovery after surgery，ERAS）的理念相契合。ERAS倡导围术期采用一系列经循证医学证实有效的优化处理措施，以最大限度地减轻患者心理和生理的应激反应，从而加速患者康复，其中促进术后康复的麻醉管理也是ERAS的重要组成部分^[3]^。要评估麻醉和手术后病人体验的干预措施的改进，需要特别强调以病人为中心的结果评估^[4]^。麻醉医生应当在围术期使用有效的方法（优化术前、术中、术后患者管理等）来降低手术伤害性刺激反应，减少并发症，提高恢复质量，从而缩短PACU停留时间和住院时间，减少医疗资源消耗，提高患者的满意度和生活质量^[5,6]^。作为麻醉科医生，我们有责任优化病人的术后体验，找出能够提供快速高质量恢复的技术，同时尽量减少发病率和日常活动恢复时间^[7]^。研究表明，围手术期麻醉药物和技术的选择是影响患者术后恢复的重要因素^[8]^。

艾司氯胺酮是是氯胺酮的右旋体，主要作用于N-甲基-D-天冬氨酸(NMDA)受体，非竞争性抑制谷胺酸对该受体的激活，并且对NMDA的阻滞有时间和刺激频率的依赖性，从而使神经元活动减弱，产生麻醉和镇痛作用，其效价是氯胺酮的2倍，镇静和镇痛作用更强，不良反应发生率低^[9,10]^。既往研究显示，氯胺酮作为NMDA受体可以提供良好的镇痛效果，和阿片类药物联用减弱中枢敏化和痛觉过敏，减少阿片类药物的用量，从而降低其相关副作用^[11,12]^。此外，艾司氯胺酮的人体清除率高、代谢快，增加了麻醉的可控性，使患者苏醒更快、更安全^[13]^。

而且，氯胺酮具有抗炎的作用，可降低人体外周血白介素IL-1β，肿瘤坏死因子TNF-α，IL-6等炎症因子的水平^[14-16]^。炎性小体（inflammasome）是一种蛋白复合体，由细胞内传感器、典型的点样受体(NLR)、接头蛋白ASC(apoptosis-associated speck-like protein containing a CARD)以及效应分子半胱氨酸依赖性的天冬氨酸水解酶-1的前体(pro-caspase-1)构成，参与多种炎症的形成，具有多种亚型，其中NLRP3是目前研究最广泛的^[17,18]^。研究证明，NLRP3炎性小体水平与疼痛、炎症、抑郁水平相关^[19-21]^。NLRP3炎性小体可促进IL-1β和IL-18的成熟和分泌，在机体的炎症反应中发挥重要的调控作用^[22]^。但对于氯胺酮或艾司氯胺酮是否能通过NLRP3炎症小体调控机体炎症水平，降低术后疼痛，改善患者术后恢复尚不清楚；而且，目前关于氯胺酮术后恢复质量的研究较少且结果不一致^[23-25]^，并缺乏观察在此类患者中使用艾司氯胺酮对患者术后早期恢复质量的影响，因此需进行观察试验。

参考文献：

[1] 中华医学会消化内镜学分会消化系旱癌内镜诊断与治疗协, 中华医学会消化病学分会消化道肿瘤协作组, 中华医学会消化内镜学分会肠道学组, et al. 中国早期结直肠癌及癌前病变筛查与诊治共识意见(2014年11月·重庆)[J]. 中华内科杂志, 2015, 4(54).

[2] Trastulli S, Cirocchi R, Listorti C, et al. Laparoscopic vs open resection for rectal cancer: a meta-analysis of randomized clinical trials[J]. Colorectal disease : the official journal of the Association of Coloproctology of Great Britain and Ireland, 2012, 14(6): e277-96.

[3] 中国加速康复外科专家组. 中国加速康复外科围手术期管理专家共识(2016)[J]. 中华外科杂志, 2016, 6(54).

[4] Shulman M, Myles P. Measuring perioperative outcome[J]. Curr Opin Anaesthesiol, 2016, 29(6): 733-738.

[5] 中国医师协会麻醉学医师分会. 促进术后康复的麻醉管理专家共识[J]. 中华麻醉学杂志, 2015, 2(35).

[6] Myles P, Williams D, Hendrata M, et al. Patient satisfaction after anaesthesia and surgery: results of a prospective survey of 10,811 patients[J]. British journal of anaesthesia, 2000, 84(1): 6-10.

[7] Murphy G, Szokol J, Greenberg S, et al. Preoperative dexamethasone enhances quality of recovery after laparoscopic cholecystectomy: effect on in-hospital and postdischarge recovery outcomes[J]. Anesthesiology, 2011, 114(4): 882-90.

[8] Lee J H. Anesthesia for ambulatory surgery[J]. Korean J Anesthesiol, 2017, 70(4): 398-406.

[9] Arendt-Nielsen L, Nielsen J, Petersen-Felix S, et al. Effect of racemic mixture and the (S+)-isomer of ketamine on temporal and spatial summation of pain[J]. British journal of anaesthesia, 1996, 77(5): 625-31.

[10] Wang J, Huang J, Yang S, et al. Pharmacokinetics and Safety of Esketamine in Chinese Patients Undergoing Painless Gastroscopy in Comparison with Ketamine: A Randomized, Open-Label Clinical Study[J]. Drug Des Devel Ther, 2019, 13: 4135-4144.

[11] 刘国凯, 黄宇光, 罗爱伦. 小剂量氯胺酮用于术后镇痛的研究及其临床价值[J]. 中华麻醉学杂志, 2003, 3(23).

[12] Bell R F, Dahl J B, Moore R A, et al. Peri-operative ketamine for acute post-operative pain: a quantitative and qualitative systematic review (Cochrane review)[J]. Acta Anaesthesiol Scand, 2005, 49(10): 1405-28.

[13] 郑旭, 顾小萍. 右旋氯胺酮临床应用的研究进展[J]. 国际麻醉学与复苏杂志, 2019, 7(40).

[14] Welters I, Feurer M, Preiss V, et al. Continuous S-(+)-ketamine administration during elective coronary artery bypass graft surgery attenuates pro-inflammatory cytokine response during and after cardiopulmonary bypass[J]. British journal of anaesthesia, 2011, 106(2): 172-9.

[15] Luggya T S, Roche T, Ssemogerere L, et al. Effect of low-dose ketamine on post-operative serum IL-6 production among elective surgical patients: a randomized clinical trial[J]. Afr Health Sci, 2017, 17(2): 500-507.

[16] Wang C, Ye Y, Chen F, et al. Posttraumatic administration of a sub-anesthetic dose of ketamine exerts neuroprotection via attenuating inflammation and autophagy[J]. Neuroscience, 2017, 343: 30-38.

[17] Cowie A, Dittel B, Stucky C. A Novel Sex-Dependent Target for the Treatment of Postoperative Pain: The NLRP3 Inflammasome[J]. Frontiers in neurology, 2019, 10: 622.

[18] Kaufmann F, Costa A, Ghisleni G, et al. NLRP3 inflammasome-driven pathways in depression: Clinical and preclinical findings[J]. Brain, behavior, and immunity, 2017, 64: 367-383.

[19] Starobova H, Nadar E I, Vetter I. The NLRP3 Inflammasome: Role and Therapeutic Potential in Pain Treatment[J]. Front Physiol, 2020, 11: 1016.

[20] Alcocer-Gomez E, Cordero M D. NLRP3 inflammasome: a new target in major depressive disorder[J]. CNS Neurosci Ther, 2014, 20(3): 294-5.

[21] Chen R, Yin C, Fang J, et al. The NLRP3 inflammasome: an emerging therapeutic target for chronic pain[J]. J Neuroinflammation, 2021, 18(1): 84.

[22] Cowie A M, Dittel B N, Stucky C L. A Novel Sex-Dependent Target for the Treatment of Postoperative Pain: The NLRP3 Inflammasome[J]. Front Neurol, 2019, 10: 622.

[23] Moro E T, Feitosa I, De Oliveira R G, et al. Ketamine does not enhance the quality of recovery following laparoscopic cholecystectomy: a randomized controlled trial[J]. Acta Anaesthesiol Scand, 2017, 61(7): 740-748.

[24] Lee J-A, Jeon Y-S, Noh H-I, et al. The Effect of Ketamine with Remifentanil for Improving the Quality of Anaesthesia and Recovery in Paediatric Patients Undergoing Middle-Ear Ventilation Tube Insertion[J]. Journal of International Medical Research, 2011, 39(6).

[25] Reed R A, Quandt J E, Brainard B M, et al. The effect of induction with propofol or ketamine and diazepam on quality of anaesthetic recovery in dogs[J]. J Small Anim Pract, 2019, 60(10): 589-593.

**二、研究目的及意义**

本研究拟观察小剂量艾司氯胺酮对腹腔镜结直肠癌根治术患者术后早期恢复质量的影响以及是否能通过NLRP3炎症小体调控炎症水平，降低术后疼痛，改善患者术后恢复，为结直肠癌根治术患者全凭静脉麻醉中合理使用艾司氯胺酮提供参考依据。

**三、研究内容（重点阐述）**

1.分组

本研究为一项前瞻性、随机对照研究，将对预实施两种不同麻醉管理方案的患者进行观察。按照随机数字表法，将患者随机分为K组（艾司氯胺酮组）和C组（对照组）。K组：全麻诱导后静脉注射艾司氯胺酮0.25mg/kg的负荷量，后持续泵注艾司氯胺酮0.12mg.kg^-1^.h^-1^直至手术结束；C组：围术期静脉注射等量生理盐水。

（以上用量均依照实际临床药物使用或专家指南使用）。

2.纳入和排除标准

（1）纳入标准

1）拟行择期全麻腹腔镜结肠癌根治术患者；

2）拟按照本研究需要观察的两种常规临床麻醉管理方案实施麻醉的患者；

3）美国麻醉医师协会（ASA）分级为I或Ⅱ级；

4）年龄18～65周岁，性别不限；

5）体重指数（BMI）18～30kg/m^2^；

6）签署麻醉知情同意书。

（2）排除标准

1）拒绝参加本研究的患者；

2）对艾司氯胺酮有禁忌症的患者（如青光眼、大血管动脉瘤等）；

3）术前使用镇静或镇痛类药物的患者；

4）严重心肺肝肾功能异常的患者；

5）有认知障碍或精神、神经系统方面疾病史不能与研究人员合作的患者。

（3）剔除标准

1）中转开腹；

2）出血量大于500ml；

3）术中出现大量皮下气肿或术后高碳酸血症短时间内无法纠正；

4）严重的术后并发症影响术后恢复质量；

5）失访；

6）手术方式为Miles。
3.研究方法

（1）术前访视

所有拟纳入研究的患者均于手术前一天进行访视，着重了解患者有无手术麻醉史，有无神经或精神系统疾病史，有无心脑肝肾疾病史，有无长期服用镇静、镇痛类药物。查阅患者相关血检验资料及影像学检查结果，对患者行体格检查，完善术前检查，确定是否存在困难气道，综合评估患者ASA分级及心功能分级。告知患者术前严格禁食8小时，禁饮2小时，告知患者及家属围术期可能出现的麻醉并发症以及意外情况，签署麻醉知情同意书。向患者及家属说明本研究的内容和临床意义，征得同意后由患者本人或其委托人签署本研究的知情同意书。

（2）麻醉前准备

患者入手术室后常规开放上肢外周静脉通路，监测心电图、血压、心率、脉搏血氧饱和度等各项生命体征。贴BIS电极片行麻醉深度监测。局麻下行桡动脉穿刺穿刺置管测量有创动脉压。

（3）麻醉管理

采用静脉快速诱导法：患者充分给氧去氮后，静脉给予依托咪酯0.2～0.3mg/kg，舒芬0.5～1.0μg/kg，顺式阿曲库铵0.15～0.3mg/kg。待达到插管条件后，行气管插管，使用容量控制模式控制呼吸，吸入氧气复合空气（氧流量1L/min，空气流量1L/min）。在超声引导下行右侧颈内静脉穿刺置管。术中调节泵注丙泊酚4～12mg.kg^-1^.h^-1^、瑞芬太尼0.05～2 ug.kg^-1^.min^-1^，维持BIS 40～60之间。间断静脉推注顺式阿曲库铵维持肌肉松弛。必要时使用血管活性药物调控血流动力学的稳定。K组诱导后给予艾司氯胺酮0.25mg/kg的负荷量，后持续泵注0.12mg.kg^-1^.h^-1^直至手术结束；C组输注等量的生理盐水。两组患者均在麻醉诱导前（T_0_）、CO_2_气腹建立后（T_1_）、术毕（T_3_）、术后1d（T_4_）四个时点抽取中心静脉血测NLRP3、IL-1β和IL-18水平，并记录相关数据。

手术结束前约30min静脉注射帕洛诺司琼0.25mg、舒芬太尼0.1～0.2μg/kg预防术后恶心呕吐和疼痛，缝合最后一层皮肤时停用所有麻醉维持用药，手术结束时连接静脉镇痛泵。镇痛泵方案为：羟考酮0.6mg/kg+帕洛诺司琼0.25mg+生理盐水=200ml，首次量0ml，背景剂量3ml/h,单次追加剂量4ml，锁定时间15min,镇痛至术后48h。

手术结束后将患者送入麻醉恢复室（Post anesthesia care unit,PACU），患者VAS评分≥4分时静脉注射羟考酮0.05～0.1mg/kg补救镇痛，在患者意识清醒、潮气量以及肌力恢复至术前水平时拔除气管导管。

4.观察指标

于麻醉诱导前基线值（T0）、CO_2_气腹建立后（T_1_）、关闭气腹后（T_2_）、术毕（T_3_）、术后1d（T_4_），由经过培训的麻醉医师分别记录如下数据：

（1）主要观察指标

术前1d、术后1d和术后3d采用15项恢复质量评分量表（15-item quality of recovery questionnaire, QoR-15）对患者进行分项评价，评估患者术后早期恢复质量

（2）次要观察指标

1）T_0_、T_1_、 T_3_、T_4_时中心静脉血NLRP3、IL-1β和IL-18水平；

2）手术时间、瑞芬太尼用量、血管活性药物使用次数和剂量；

3）T_0_-T_3_时 MAP、HR血流动力学水平；

4）PACU期间羟考酮使用次数和剂量、自主呼吸恢复时间(停药至自主呼吸恢复时间)、反应时间（停药至口头指令有反应时间）、拔管时间（停药至拔除气管导管时间）；

5) 术后4、8、12、24h 、48h静息和运动时VAS疼痛评分；术后24h及48h镇痛泵按压次数、羟考酮消耗量以及是否使用额外镇痛药物；

6）术后住院时间，术后肠道功能复时间(气管导管拔出至第1次排气时间)，进食进水时间(气管导管拔出至第1次进水或进食时间)、下床活动时间(气管导管拔出至第1次下床站立或行走时间)、术后24h内恶心、呕吐、幻觉、做梦等不良反应发生情况。

5.研究终止

发生以下情况时，本研究可能会被中止：

（1）研究期间发生严重不良事件；

（2）研究方案存在严重错误；

（3）研究资金不足；

（4）行政主管机构要求停止研究。

6.质量控制

（1）研究者

1) 所有参研人员均将接受研究方案培训；

2) 研究人员严格按照纳入标准和排除标准筛选病例；

3) 研究人员需要认真如实填写研究数据；

4) 为尽可能减少偏倚，所有纳入病例术后随访均由对分组情况不知情同一

研究人员承担。

（2）受试者

1) 在入选受试者时，研究人员将向其详细介绍本研究方案，尤其是可能面临

的风险及受益；

2) 必须获得受试者或其法定代理人签署的知情同意书；

3) 受试者可在研究期间随时退出研究且不需要任何理由。

7.统计方法

根据之前的研究，我们假设本研究中试验组和对照组术后24hQoR-15评分最小临床差异为8分。假设检验水准α取0.05，检验效能Power(1-β)取0.8，每组预计需要42例患者，考虑到10%的退出率，预期需要每组入选46例患者，本研究一共需要92例患者。

采用SPSS21.0软件进行分析，正态分布的计量资料以均数±标准差（‾x±s）表示，组间比较采用独立样本t检验；偏态分布的计量资料以中位数（*M*）和四分位数间距M(P25, P75)表示，采用Mann-Whitney U检验；计数资料以例（%）表示，采用卡方检验、Fisher’s精确检验进行组间比较；等级资料的组间比较采用秩和检验；采用重复测量方差分析对各个时间点的数据进行比较。双侧检验P<0.05被认为差异有统计学意义。

1. **预期研究结果**

小剂量艾司氯胺酮可以改善腹腔镜结肠癌根治术患者术后早期恢复质量；并且能通过NLRP3炎症小体调控炎症水平，降低术后疼痛，改善患者术后恢复，为结直肠癌根治术患者全凭静脉麻醉中合理使用艾司氯胺酮提供参考依据。
