# Supplementary material for: Intraoperative intravenous low-dose esketamine improves quality of early recovery after laparoscopic radical resection of colorectal cancer: A prospective, randomized controlled trial
Source: PLoS One. 2023 Jun 2;18(6):e0286590. doi: 10.1371/journal.pone.0286590 (PMC10237502; doi:10.1371/journal.pone.0286590)
Supplement: S3 File — (DOCX) [file pone.0286590.s003.docx]

Study protocol of the effect of low-dose esketamine on the quality of early recovery in patients undergoing laparoscopic radical resection of colorectal cancer

**Applicant :** Yanqiu Ai

**Tel :** +8613607690334

**Department :** Department of Anesthesiology, Pain and Perioperative Medicine, The First Affiliated Hospital of Zhengzhou University

**Contact :** Ying Xu

**Tel :** +8613523550017

1. **Basis for project approval**

Colorectal cancer (CRC) is a common clinical malignant tumor. With the change of living habits and diet structure, the incidence of CRC has been increasing in recent years^[1]^. Currently, CRC is mainly treated by comprehensive methods based on surgical resection. Compared with traditional open surgery, laparoscopic radical surgery is minimally invasive and has fewer postoperative complications, which has become a common surgical method in clinical practice^[2]^. To a certain extent, it accelerates postoperative recovery, which is consistent with the concept of enhanced recovery after surgery (ERAS). ERAS advocates the use of a series of optimized perioperative treatment measures confirmed by evidence-based medicine to minimize the psychological and physiological stress response of patients, so as to accelerate the recovery of patients^[3]^. Among them, anesthesia management to promote postoperative rehabilitation is also an important part of ERAS. Improvements in interventions to evaluate patient experience after anesthesia and surgery require a special emphasis on patient-centered outcome assessment^[4]^. Anesthesiologists should use effective methods during the perioperative period (optimizing preoperative, intraoperative and postoperative patient management, etc.) to reduce the noxious stimulus response to surgery and complications, and improve the quality of recovery, so as to shorten the length of stay in PACU and hospital, reduce the consumption of medical resources, and improve the satisfaction and quality of life of patients^[5,6]^. As anesthesiologists, we have a responsibility to optimize the postoperative experience and identify techniques that provide rapid, high-quality recovery while minimizing morbidity and time to return to daily activities^[7]^. The choice of perioperative anesthetic drugs and techniques is an important factor affecting the postoperative recovery of patients^[8]^.

Esketamine is the dextroversion of ketamine, mainly acts on N-methyl-D-aspartate (NMDA) receptor, non-competitively inhibits the activation of the receptor by glutamine, and has a time-dependent and frequency-dependent block of NMDA, so as to weaken neuronal activity and produce anesthetic and analgesic effects^[9]^. And, its potency is twice that of ketamine, which leads to stronger sedative and analgesic effects, and a lower incidence of adverse reactions than ketamine^[10]^. Previous studies have shown that ketamine, as an NMDA receptor, can provide good analgesic effect, and reduce central sensitization and hyperalgesia when combined with opioids, thereby reducing the dosage of opioids and their related side effects^[11,12]^. Moreover, esketamine has a high clearance rate and rapid metabolism in human body, which increases the controllability of anesthesia, and makes patients awake faster and safer^[13]^.

Ketamine has an anti-inflammatory effect, which can reduce the levels of IL-1β, TNF α, IL-6 and other inflammatory factors in human peripheral blood^[14-16]^. The inflammasome is a protein complex that comprises an intracellular sensor, typically a Nod-like receptor (NLR), the precursor procaspase-1, and the adaptor ASC; and has a variety of subtypes, among which NLRP3 is the most widely studied^[17,18]^. Studies have proved that NLRP3 inflammasome is related to the level of pain, inflammation, and depression^[19-21]^. NLRP3 inflammasome can promote the maturation and secretion of IL-1β and IL-18, and play an important regulatory role in the inflammatory response of the body^[22]^. However, whether ketamine or esketamine can regulate the level of inflammation in the body through NLRP3 inflammasome, reduce postoperative pain, and improve postoperative recovery of patients is still unclear. At present, there are few ketamine studies on the quality of recovery with inconsistent results, and there is a lack of research on the effect of esketamine on the early quality of recovery^[23-25]^. Therefore, this trial is required.

**References**

[1] 中华医学会消化内镜学分会消化系旱癌内镜诊断与治疗协, 中华医学会消化病学分会消化道肿瘤协作组, 中华医学会消化内镜学分会肠道学组, et al. 中国早期结直肠癌及癌前病变筛查与诊治共识意见(2014年11月·重庆)[J]. 中华内科杂志, 2015, 4(54).

[2] Trastulli S, Cirocchi R, Listorti C, et al. Laparoscopic vs open resection for rectal cancer: a meta-analysis of randomized clinical trials[J]. Colorectal disease : the official journal of the Association of Coloproctology of Great Britain and Ireland, 2012, 14(6): e277-96.

[3] 中国加速康复外科专家组. 中国加速康复外科围手术期管理专家共识(2016)[J]. 中华外科杂志, 2016, 6(54).

[4] Shulman M, Myles P. Measuring perioperative outcome[J]. Curr Opin Anaesthesiol, 2016, 29(6): 733-738.

[5] 中国医师协会麻醉学医师分会. 促进术后康复的麻醉管理专家共识[J]. 中华麻醉学杂志, 2015, 35(2): 141-148.

[6] Myles P, Williams D, Hendrata M, et al. Patient satisfaction after anaesthesia and surgery: results of a prospective survey of 10,811 patients[J]. British journal of anaesthesia, 2000, 84(1): 6-10.

[7] Murphy G, Szokol J, Greenberg S, et al. Preoperative dexamethasone enhances quality of recovery after laparoscopic cholecystectomy: effect on in-hospital and postdischarge recovery outcomes[J]. Anesthesiology, 2011, 114(4): 882-90.

[8] Lee J H. Anesthesia for ambulatory surgery[J]. Korean J Anesthesiol, 2017, 70(4): 398-406.

[9] Arendt-Nielsen L, Nielsen J, Petersen-Felix S, et al. Effect of racemic mixture and the (S+)-isomer of ketamine on temporal and spatial summation of pain[J]. British journal of anaesthesia, 1996, 77(5): 625-31.

[10] Wang J, Huang J, Yang S, et al. Pharmacokinetics and Safety of Esketamine in Chinese Patients Undergoing Painless Gastroscopy in Comparison with Ketamine: A Randomized, Open-Label Clinical Study[J]. Drug Des Devel Ther, 2019, 13: 4135-4144.

[11] 刘国凯, 黄宇光, 罗爱伦. 小剂量氯胺酮用于术后镇痛的研究及其临床价值[J]. 中华麻醉学杂志, 2003, 3(23).

[12] Bell R F, Dahl J B, Moore R A, et al. Peri-operative ketamine for acute post-operative pain: a quantitative and qualitative systematic review (Cochrane review)[J]. Acta Anaesthesiol Scand, 2005, 49(10): 1405-28.

[13] 郑旭, 顾小萍. 右旋氯胺酮临床应用的研究进展[J]. 国际麻醉学与复苏杂志, 2019, 40(7): 674-677.

[14] Welters I, Feurer M, Preiss V, et al. Continuous S-(+)-ketamine administration during elective coronary artery bypass graft surgery attenuates pro-inflammatory cytokine response during and after cardiopulmonary bypass[J]. British journal of anaesthesia, 2011, 106(2): 172-9.

[15] Luggya T S, Roche T, Ssemogerere L, et al. Effect of low-dose ketamine on post-operative serum IL-6 production among elective surgical patients: a randomized clinical trial[J]. Afr Health Sci, 2017, 17(2): 500-507.

[16] Wang C Q, Ye Y, Chen F, et al. Posttraumatic administration of a sub-anesthetic dose of ketamine exerts neuroprotection via attenuating inflammation and autophagy[J]. Neuroscience, 2017, 343: 30-38.

[17] Cowie A M, Dittel B N, Stucky C L. A Novel Sex-Dependent Target for the Treatment of Postoperative Pain: The NLRP3 Inflammasome[J]. Front Neurol, 2019, 10: 622.

[18] Kaufmann F, Costa A, Ghisleni G, et al. NLRP3 inflammasome-driven pathways in depression: Clinical and preclinical findings[J]. Brain, behavior, and immunity, 2017, 64: 367-383.

[19] Starobova H, Nadar E I, Vetter I. The NLRP3 Inflammasome: Role and Therapeutic Potential in Pain Treatment[J]. Front Physiol, 2020, 11: 1016.

[20] Alcocer-Gomez E, Cordero M D. NLRP3 inflammasome: a new target in major depressive disorder[J]. CNS Neurosci Ther, 2014, 20(3): 294-5.

[21] Chen R, Yin C, Fang J, et al. The NLRP3 inflammasome: an emerging therapeutic target for chronic pain[J]. J Neuroinflammation, 2021, 18(1): 84.

[22] Cowie A, Dittel B, Stucky C. A Novel Sex-Dependent Target for the Treatment of Postoperative Pain: The NLRP3 Inflammasome[J]. Frontiers in neurology, 2019, 10: 622.

[23] Moro E T, Feitosa I, De Oliveira R G, et al. Ketamine does not enhance the quality of recovery following laparoscopic cholecystectomy: a randomized controlled trial[J]. Acta Anaesthesiol Scand, 2017, 61(7): 740-748.

[24] Lee J-A, Jeon Y-S, Noh H-I, et al. The Effect of Ketamine with Remifentanil for Improving the Quality of Anaesthesia and Recovery in Paediatric Patients Undergoing Middle-Ear Ventilation Tube Insertion[J]. Journal of International Medical Research, 2011, 39(6).

[25] Reed R A, Quandt J E, Brainard B M, et al. The effect of induction with propofol or ketamine and diazepam on quality of anaesthetic recovery in dogs[J]. J Small Anim Pract, 2019, 60(10): 589-593.

**2. Objective and implication**

This study aims to observe the effect of low-dose esketamine on the early postoperative quality of recovery in patients undergoing laparoscopic radical resection of colorectal cancer and whether it can regulate the level of inflammation through NLRP3 inflammasome, reduce postoperative pain, and improve postoperative recovery of patients, so as to provide reference for the rational use of esketamine in total intravenous anesthesia.

**3. Content of the study**

3.1 Grouping

This is a prospective, randomized controlled trial. Patients will be randomly assigned in a 1:1 ratio to esketamine group (K group) and control group (C group) using a random number table method. In the K group, a loading dose of 0.25mg/kg of esketamine will be given after anesthesia induction, and then a rate of 0.12mg/kg/h will be continuous infusion until the end of surgery. The equal volume of normal saline will be infused in the C group.

(The above doses will be used in accordance with the actual clinical use of drugs or guidelines)

3.2 Inclusion and exclusion criteria

(1) Inclusion criteria

1) Patients scheduled for elective laparoscopic radical resection of colon cancer under general anesthesia;

2) Patients who will be to be anesthetized according to the two clinical anesthesia management protocols ;

3) American Society of Anesthesiologists (ASA) physical status Ⅰ-Ⅱ;

4) Patients aging from 18 to 65 years, no gender limit;

5) BMI of 18-30 kg/m^2^;

6) Written informed consent for anesthesia.

(2) Exclusion criteria

1) Refusal to participate in the study;

2) Contraindications to esketamine, such as glaucoma, large vessel aneurysm, etc.;

3) Preoperative use of sedative or analgesic drugs;

4) Severe cardiopulmonary, liver and kidney dysfunction;

5) Cognitive impairment or a history of psychiatric or neurological disorders.

(3) Elimination

1) Conversion to laparotomy;

2) The bleeding volume was more than 500ml;

3) A large amount of subcutaneous emphysema during the operation or hypercapnia after operation could not be corrected in a short time;

4) Severe postoperative complications affected the quality of postoperative recovery;

5) Patients are lost follow-up;

6) The surgical procedure is Miles.

3.3 Methods

(1) Preoperative visit

All patients to be included in the study will be interviewed before surgery, focusing on whether the patient had a history of anesthesia, neurological or psychiatric system diseases, heart, brain, liver and kidney diseases, and long-term use of sedative and analgesic drugs. The relevant blood test data and imaging examination results of the patients will be reviewed. The physical examination of the patients will be performed, and the preoperative examination will be improved to determine whether there will be a difficult airway. The ASA classification and cardiac function classification of the patients will be comprehensively evaluated. The patients will be told to strictly fast for 8 hours and drink for 2 hours before surgery. The patients and their families will be informed of the possible anesthetic complications and accidents during the perioperative period, and the informed consent of anesthesia will be signed. The content and clinical significance of this study will be explained to the patients and their families. Informed consent for this study will be signed by the patients or their proxies after consent was obtained.

(2) Preparation before anesthesia

After patients enter the operation room, peripheral venous access will be opened and electrocardiogram (ECG), non-invasive blood pressure (NBP), heart rate (HR), pulse oxygen saturation (SpO_2_), and the bispectral index (BIS) will be monitored. The radial artery and right internal jugular vein will be punctured and catheterized under local anesthesia.

(3) Management of anesthesia

A rapid intravenous induction method will be used. After adequate preoxygenation, all patients will be induced for general anesthesia by intravenous injection of 0.2-0.3mg/kg etomidate, 0.5-1.0μg/kg sufentanil, 0.15-0.3mg/kg cisatracurium, and will maintain with 4-12mg/kg/h propofol and 0.05-2ug/kg/min remifentanil after intubation. The BIS value of the subjects will be maintained between 40 and 60 by adjusting the infusion rate of propofol during surgery. Oxygen and air inhalation flow will be set as 1L/min, V_T_ 6-8 ml/kg,RR 12-16 times/min,I:E=1:2 and P_ET_CO_2_ 35-45 mmHg（1mmHg=0.133kPa）. Intermittent intravenous injection of cisatracurium will be used to maintain muscle relaxation during surgery. Vasoactive drugs will be used to regulate hemodynamic stability when necessary. In the K group, a loading dose of 0.25mg/kg of esketamine will be given after anesthesia induction, and then a rate of 0.12mg/kg/h will be continuous infusion until the end of surgery. The equal volume of normal saline will be infused in the C group. The levels of NLRP3, IL-1β and IL-18 will be measured before induction (T0), after establishment of CO2 pneumoperitoneum (T1), at the end of surgery (T3) and on the first day after surgery (T4).

Palonosetron, 0.25mg, will be intravenously injected to prevent nausea and vomiting, and sufentanil, 0.1-0.2 μg/kg, will be used for analgesia about 30 minutes before the end of surgery. All anesthesia maintenance drugs will be discontinued after finishing skin closure. At the end of surgery, all patients in the two groups will be given a patient-controlled intravenous analgesia pump (PCIA,200ml) consisting of oxycodone 0.6mg/kg and palonosetron 0.25mg for 48h after surgey. The background infusion rate will be 3ml/h, single additional dose will be 4ml, the locking time will be 15min, and there will be no initial infusion dose.

After the operation,all patients will be transferred to the post anesthesia care unit (PACU). During the stay in PACU, oxycodone 0.05-0.1 mg/kg will be injected intravenously for rescue analgesia when the VAS score will be ≥ 4. The endotracheal tube will be removed when the patient become conscious and the tidal volume and muscle strength have returned to preoperative levels.

3.4 Outcomes

(1) Primary outcome

The primary outcome of this study is quality of recovery at 24 h after surgery, and it is assessed using the QoR-15 scale. The QoR-15 will be evaluated at three timepoints: before(T_before_), 24h(T_24h_) and 72h(T_72h_) after surgery.

(2) Secondary outcomes

1) The levels of NLRP3, IL-1β and IL-18 will be recorded at T0, T1, T3 and T4;

2) The duration of sugery, consumption of remifentanil, cisatracurium [dosage](javascript:;), application of vasoactive drugs;

3) MAP (mean arterial pressure) and HR will be recorded at before induction (T_0_), after the establishment of carbon dioxide pneumoperitoneum (T_1_), after closing the carbon dioxide pneumoperitoneum (T_2_), and at the end of surgery (T_3_);

4) Rescue analgesia during PACU, respiratory recovery time (the duration of time from withdrawal of anesthetics to return of spontaneous breathing), response time (the duration of time from withdrawal of anesthetics to a verbal command), extubation time (the duration of time from withdrawal of anesthetics to extubation).

5) VAS pain scores at rest (VAS-R) and on coughing (VAS-M) at 4h, 8h, 12h, 24h and 48h postoperatively; times of effective PCIA press at 24h and 48h after operation, the consumption of oxycodone, the use of additional analgesic drugs.

6) Hospital stay durations, intestinal function recovery time (the duration of time from extubation to the first exhaust time), time of first eating or drinking (the duration of time from extubation to the first drinking or eating time), ambulation time (the duration of time from extubation to the first bed time standing or walking), and adverse events such as nausea, vomiting, dizziness, hallucination and nightmare,etc.

3.5 Termination of study

The study can be stopped if the following conditions occur:

(1) Serious adverse events occurr during the study;

(2) There are serious protocol errors;

(3) Insufficient research funding;

(4) The administrative authorities request that the study is stopped.

3.6 Quality control

(1) Researchers

1) All participants will be trained in the protocol;

2) The investigators will screen the cases strictly according to the inclusion and exclusion criteria;

3) Researchers fill in data carefully and truthfully;

4) In order to minimize bias, all the included patients will be followed uo by the same researcher who is unaware of the grouping.

(2) Subjects

1) When subjects are enrolled, they will be given a detailed introduction to the study protocol by the researcher, especially risks and benefits;

2) Written informed consent will be signed by the subjects or their legal representatives;

3) Participants can withdraw from the study at any time without any reason.

3.7 Statistical analysis

The primary outcome of this study was the quality of recovery score (QoR-15) at 24 h postoperatively. The minimal clinically important difference (MCID) in the QoR-15 scale was 8.0. According to previous research, the sample size calculation showed that 42 subjects in each group were required to achieve 80% power with an α of 0.05. To allow for a dropout rate of 10%, we finally enrolled 46 subjects in each group in this study.

The SPSS21.0 software (IBM) was used for statistical analysis. Shapiro-Wilk test was used to test the normality of the data. Data with normal distribution were presented as mean and standard deviation (SD), and comparisons between groups were analyzed using A Student’s *t*-test. Datas collected at multiple time points were analyzed using repeated measures analysis of variance (ANOVA). Data with non-normal distribution were expressed as median (interquartile range). The Mann-Whitney *U* test was used for intergroup comparisons. The categorical data were shown as numbers (%) and analyzed using the chi-square test or Fisher’s exact test as needed. A two-sided test *P*-value less than 0.05 was considered statistically significant.

**4. Anticipated study results**

Low-dose esketamine can improve the early postoperative quality of recovery in patients undergoing laparoscopic radical resection of colon cancer. It can regulate the level of inflammation through NLRP3 inflammasome, reduce postoperative pain, improve postoperative recovery of patients, and provide reference for the rational use of esmketamine in total intravenous anesthesia in patients undergoing radical resection of colorectal cancer.
