# Supplementary material for: Intraoperative intravenous low-dose esketamine improves quality of early recovery after laparoscopic radical resection of colorectal cancer: A prospective, randomized controlled trial
Source: PLoS One. 2023 Jun 2;18(6):e0286590. doi: 10.1371/journal.pone.0286590 (PMC10237502; doi:10.1371/journal.pone.0286590)
Supplement: Attachment — Submitted filename: Comments on PONE.docx [file pone.0286590.s004.docx]

Comments on PONE-D-22-33531

Thank you for inviting me to revise this manuscript. The authors discussed a clinically relevant topic. The manuscript is generally well written, but I have some comments.

Abstract

- VAS was mentioned without identification
- You mentioned group k and group C in the results without identification to which group they referred to. Please identify the group name in the method part of the abstract e.g.: “*were randomized to Esketamine (K group) and non-eskatamine groups (C group)*” however; using a self-explaining name for the group is better
- No need to mention all the study outcomes in the abstract specially when their results were not mentioned in the results section of the abstract
- The conclusion in the abstract should reflect the information mentioned in it, and since you did not mention any results related to adverse events in the results part, you should not mention that the drug “*and did not increase postoperative adverse reaction*”
- Please change colorectal patients to patients with colorectal cancer

Introduction

- In the first paragraph, you need to explain the operative related complication that could be improved by the study drug. Otherwise, it is better to remove the sentence starting with “However, the establishment…” as the role of the study drug is not to improve or impact those complications. You may consider other complications, such as inadequate pain management and use of opioids, which could be affected by the study drug.
- In the second paragraph, mention some of the adverse effect of ketamine in comparison to the esketamine.
- In the same paragraph, you should break down the last sentence.

Methods

- The blinding is not clear, you described the rate in the esketamine group as 0.25mg/kg of esketamine as bolus, and then a rate of 0.12mg/kg/h, and that equal volume of normal saline was infused in the C group. please clarify how is equal volume was calculated
- In the secondary outcomes please remove “etc” and enumerate the recorded complications.

Sample size

Please provide more details regarding the assumed SD for the sample size calculation and the used program, ref 18 is not relevant to your study population

Statistical analysis

Please change:

“A Student t-test” to “the Student t-test”

“Datas” to Data

(interquartile range) to (quartiles)

Add how the normality of data was checked

**Results**

In the first paragraph last sentence please edit to “were not significantly different”

Discussion

P 9 third paragraph: S-ketamine to esketamine

Some grammatical mistakes: “leaded” should be “led”
