# Supplementary material for: Intraoperative intravenous low-dose esketamine improves quality of early recovery after laparoscopic radical resection of colorectal cancer: A prospective, randomized controlled trial
Source: PLoS One. 2023 Jun 2;18(6):e0286590. doi: 10.1371/journal.pone.0286590 (PMC10237502; doi:10.1371/journal.pone.0286590)
Supplement: Attachment — Submitted filename: Response to Reviewers.docx [file pone.0286590.s005.docx]

Detailed Response to Reviewers

Reference: PONE-D-22-33531

Title: Intraoperative intravenous low-dose esketamine improves quality of early recovery after laparoscopic radical resection of colorectal cancer: A prospective, randomized controlled trial

Journal title: PLOS ONE

Authors: Ying Xu, Long He, Shaoxuan Liu, Chaofan Zhang, Yanqiu Ai

Dear Editor:

Thank you for your useful comments on our manuscript. We wish to give a sincere gratitude to referees for reviewing our paper carefully. We apologize for any inconveniences caused by these errors. We have modified the manuscript accordingly, and the specific modifications are listed point by point below.

Reviewer: 1

Abstract

- VAS was mentioned without identification

Thank you for the suggestion. This section has been removed due to the third comment “No need to mention all the study outcomes”. However, VAS was mentioned with identification on the page 3 “*The visual analogue scale (VAS) was used to assess pain intensity;*”.

- You mentioned group k and group C in the results without identification to which group they referred to. Please identify the group name in the method part of the abstract e.g.: “*were randomized to Esketamine (K group) and non-eskatamine groups (C group)*” however; using a self-explaining name for the group is better

Thank you for the suggestion. We have corrected it in the method part of the abstract : “*were randomly assigned to either the esketamine (K group) or non-eskatamine (C group) group.*”.

- No need to mention all the study outcomes in the abstract specially when their results were not mentioned in the results section of the abstract

Thank you for the suggestion. Secondary outcomes in our study has been removed in the abstract.

- The conclusion in the abstract should reflect the information mentioned in it, and since you did not mention any results related to adverse events in the results part, you should not mention that the drug “*and did not increase postoperative adverse reaction*”

Thank you for the suggestion. We added that “*The postoperative adverse events were comparable between the two groups.*” in the result part.

- Please change colorectal patients to patients with colorectal cancer

Thank you very much for the suggestion. We have corrected it to patients with colorectal cancer.

Introduction

- In the first paragraph, you need to explain the operative related complication that could be improved by the study drug. Otherwise, it is better to remove the sentence starting with “However, the establishment…” as the role of the study drug is not to improve or impact those complications. You may consider other complications, such as inadequate pain management and use of opioids, which could be affected by the study drug.

Thank you for the suggestion. We have revised the sentence to “*However, pain after laparoscopic surgery is a common issue that may* interfere with *discharge and recovery.*”.

- In the second paragraph, mention some of the adverse effect of ketamine in comparison to the esketamine.

Thank you for the suggestion. We have added this sentence “*Because of the dose-dependent side effects of ketamine, esketamine has a lower likelihood of adverse reactions (such as psychedelic effects).*”.

- In the same paragraph, you should break down the last sentence.

Thank you for the suggestion. We have broken down the last sentence that “*The 15-item Quality of Recovery scale (QoR-15) is a patient-centered outcome measure used to assess patients’ health status following surgery and anesthesia. Furthermore, the QoR-15 is dependable, valid and has high clinical acceptability.*”.

Methods

- The blinding is not clear, you described the rate in the esketamine group as 0.25mg/kg of esketamine as bolus, and then a rate of 0.12mg/kg/h, and that equal volume of normal saline was infused in the C group. please clarify how is equal volume was calculated

Thank you for the suggestion. We have clarified this with “*Esketamine, 50 mg, was diluted to a total of 50 ml with normal saline solution. Both esketamine and normal saline were drawn into identical 50-ml syringes by the same anesthesia nurse.*”.

- In the secondary outcomes please remove “etc” and enumerate the recorded complications.

Thank you for the suggestion. We have removed “etc” and enumerate the recorded complications “*pruritis*”.

Sample size

Please provide more details regarding the assumed SD for the sample size calculation and the used program, ref 18 is not relevant to your study population

Thank you for the suggestion. We have provided more details on the sample size calculation :“*The primary outcome of this study was the quality of recovery score (QoR-15) at 24 h postoperatively.* *PASS 15.0 was used to calculate the estimated sample size based on the primary outcome. The minimal clinically important difference (MCID) in the QoR-15 scale was 8.0.* *According to previous research and preliminary findings,* *the total QoR-15 score at 24 h postoperatively in the esketamine group was 114 ± 16, compared to 102 ± 22 in the control group.* *A sample size of 42 subjects in each group was calculated using the preliminary study data, assuming Type Ⅰ error (α)＝0.05 and Type Ⅱ error (β)＝0.2.* *To allow for a dropout rate of 10%, we finally enrolled 46 subjects in each group in this study.*”.

Statistical analysis

Please change:

“A Student t-test” to “the Student t-test”

Thank you for the suggestion. We have corrected it to the Student’s t-test.

“Datas” to Data

Thank you for the suggestion. We have corrected it.

(interquartile range) to (quartiles)

Thank you for the suggestion. We have corrected it to (quartiles).

Add how the normality of data was checked

Thank you for the suggestion. It’s in the second sentence of this part “The *Shapiro-Wilk test was used to test the data’s normality.*”

**Results**

In the first paragraph last sentence please edit to “were not significantly different”

Thank you for the suggestion. We have corrected it to “*were not significantly different*”

Discussion

P 9 third paragraph: S-ketamine to esketamine

Thank you very much for the suggestion. We have corrected it to esketamine.

Some grammatical mistakes: “leaded” should be “led”

Thank you very much for the suggestion. We have corrected it.

Reviewer: 2

1.The article's writing quality has to be further enhanced because it does not adhere to the magazine's standards for statistical analysis methods, tables, charts, and other elements.

Thank you for the suggestion. We have made appropriate changes according to the magazine’s standards.

2.To make the text more readable, the authors should modify the language. I suggest the authors hire a qualified native English editor.

Thank you for the suggestion. We have hired one or more of the highly qualified native English speaking editors at Springer Nature Author Services (SNAS) to modify the language. The Editing Certificate has been uploaded as an attachment.

3.To ensure that readers fully grasp what the researchers looked at, the technical information should be enlarged and explained.

Thank you for the suggestion. We have made some changes to this.

4.Page 1 Methods, VAS, PACU, etc., Abbreviations should be noted in full English whenever they first appear.

Thank you for the suggestion. This section has been removed because we made appropriate changes to the abstract. And, abbreviations have been noted in full English in the Methods part on Page 4.

5.Please update the ‘Methods’ section of the abstract to include details of the methods, results.

Thank you for the suggestion. We have updated the ‘Methods’ section of the abstract “*This* *was a prospective, randomized controlled trial.* *Ninety-two patients undergoing laparoscopic radical resection of colorectal cancer* *were randomly assigned to either the esketamine (K group) or non-eskatamine (C group) group. After anesthesia induction, a loading dose of 0.25 mg/kg was administered, followed by continuous infusion at a rate of 0.12 mg/kg/h until the end of surgery in the K group. In the C group, an equivalent volume of normal saline was infused. The primary outcome was quality of recovery at 24 h after surgery, as measured by the Quality of Recovery-15 (QoR-15) scale. The QoR-15 was evaluated at three timepoints: before (T_before_), 24 h (T_24h_) and 72 h (T_72h_) after surgery.* ”.

6.The grouping of methods and results in the abstract is not clearly described.

Thank you very much for the suggestion. We have corrected it. The grouping of methods in the abstract is described as “*Ninety-two patients undergoing laparoscopic radical resection of colorectal cancer were randomly assigned to either the esketamine (K group) or non-eskatamine (C group) group.*”. And, the results in the abstract is described as “*A total of 88 patients completed this study. The total QoR-15 scores in K group (n=45) were higher than in the C group (n=43) at 24 h: 112.33 ± 8.79 vs. 103.93 ± 9.03 (P = 0.000) and at 72h: 118.73 ± 7.82 vs. 114.79 ± 7.98 (P = 0.022). However, the differences between the two groups only had clinical significance at 24h after surgery. Among the five dimensions of the QoR-15, physical comfort (P = 0.003) ,emotional state (P = 0.000), and physical independence (P = 0.000) were significantly higher at 24h in the K group, and physical comfort (P = 0.048) was higher at 72h in the K group. The postoperative adverse events were comparable between the two groups.*”.

7.Please update the ‘Introduction’ section of the therefore.

Thank you for the suggestion. We have made some appropriate changes to the ‘Introduction’ section.

8.Why was the age range for the study's participants set at 18 to 65 years old? I don't think this age structure makes sense; if you can, please cite some references.

Thank you for the suggestion. We are very sorry for your confusion. According to the medicine specification of esketamine (SFDA Approval No.: H20193336, Jiangsu Hengrui Pharmaceutical Co Ltd), the use of esketamine is not clear in children and elderly patients. Therefore, the age range for the study's participants was set at 18 to 65 years old.

9.‘Measures related to the blood specimens in the study protocol were not shown in the manuscript because of improper storage of the blood specimens. ’ ? It appeared out of nowhere. I don't comprehend.

Thank you for the suggestion. We are very sorry for your confusion. The sentence is to illustrate the item 6b ‘Any changes to trial outcomes after the trial commenced, with reasons’ of the CONSORT 2010 checklist. Due to improper storage of blood samples, the results associated with them could not be shown in the manuscript. We have removed it.

10.Please provide information to the "Sample size calculation" section.

Thank you for the suggestion. We have provided more information on the sample size calculation :“*The primary outcome of this study was the quality of recovery score (QoR-15) at 24 h postoperatively. PASS 15.0 was used to calculate the estimated sample size based on the primary outcome. The minimal clinically important difference (MCID) in the QoR-15 scale was 8.0. According to previous research and preliminary findings, the total QoR-15 score at 24 h postoperatively in the esketamine group was 114 ± 16, compared to 102 ± 22 in the control group. A sample size of 42 subjects in each group was calculated using the preliminary study data, assuming Type Ⅰ error (α)＝0.05 and Type Ⅱ error (β)＝0.2. To allow for a dropout rate of 10%, we finally enrolled 46 subjects in each group in this study.*”.

11.Figure 1, Fig 1......, Verify the syntax, unify the names, and review the entire document.

Thank you very much for the suggestion. We have verified the the syntax, unified the names, and reviewed the entire document.

12.Table 1, etc., We preserve how many decimal places? Please consult the literature; all statistical findings must adhere to a single standard.

Thank you very much for the suggestion. We have consulted the literature on decimal places and modified it.

13.Table 2, 3, 4, It is appropriate for the appropriate position.

Thank you very much for the suggestion.

Reviewer: 3

1. You cite reference 18 as giving the sample size, but it's not clear to me that this trial had the same primary outcome, the same patient population, and the same variability that you had in your study. Can you justify using someone else's sample size computation? And if they already did the same study you did, why did you have to redo it?

Thank you for the suggestion. We apologize for the inappropriate expression we made in the manuscript. Our study had the same primary outcome as reference 18, but the patient population and primary intervention are different. We have provided more details on the sample size calculation :“*The primary outcome of this study was the quality of recovery score (QoR-15) at 24 h postoperatively. PASS 15.0 was used to calculate the estimated sample size based on the primary outcome. The minimal clinically important difference (MCID) in the QoR-15 scale was 8.0. According to previous research and preliminary findings, the total QoR-15 score at 24 h postoperatively in the esketamine group was 114 ± 16, compared to 102 ± 22 in the control group. A sample size of 42 subjects in each group was calculated using the preliminary study data, assuming Type Ⅰ error (α)＝0.05 and Type Ⅱ error (β)＝0.2. To allow for a dropout rate of 10%, we finally enrolled 46 subjects in each group in this study.*”.

2. So many p-values, and no adjustment for multiple testing. Yet you say 0.048 is significant. Without an adjustment for multiple testing, I don't believe your "significance" statements.

Thank you for the suggestion. We apologiz for the inappropriate expression. What we're trying to say is that 0.048 is a statistical difference and physical comfort (42.31 ± 4.55 vs. 40.26 ± 5.04, *P* = 0.048) was higher at 72 h in the K group. We have corrected it.

3. How do I know your sample size was adequate? It would be helpful to add to the discussion whether the assumptions underlying these computations were actually realized in the trial, but then you don't state the assumptions because they are taken from a different paper.

Thank you for the suggestion. In response to your question, we have conducted the post hoc analysis. Based on the mean and standard deviation (SD) of the two groups, the effect size ^d^Cohen calculated was 0.943 by the following website: <https://www.psychometrica.de/effect_size.html> Next, G*Power 3.1.9.7 was used to calculate the Power (1－βerr prob), which was 0.994. It is greater than 0.8. Therefore, our sample was adequate.

4. Anytime you use repeated measures ANOVA, you need to give the assumptions of the model, and how you verified those assumptions (e.g., diagnostics).

Thank you for the suggestion. We apologize for the inaccurate expression. We have corrected it to “*Data with normal distribution at multiple time points were analyzed using repeated measures analysis of variance (ANOVA).*”.
