# Supplementary material for: Intraoperative intravenous low-dose esketamine improves quality of early recovery after laparoscopic radical resection of colorectal cancer: A prospective, randomized controlled trial
Source: PLoS One. 2023 Jun 2;18(6):e0286590. doi: 10.1371/journal.pone.0286590 (PMC10237502; doi:10.1371/journal.pone.0286590)
Supplement: Attachment — Submitted filename: Reviewer3.docx [file pone.0286590.s006.docx]

Title: Intraoperative intravenous low-dose esketamine improves quality of early recovery after laparoscopic radical resection of colorectal cancer: A prospective, randomized controlled trial .

Thank you for the chance of reviewing this interesting manuscript. This study aimed to assess the effects of intraoperative intravenous low-dose esketamine on the quality of early recovery in patients undergoing laparoscopic radical resection of CRC using the QoR-15 scale. The study design has exciting clinical guiding value. Especially for the management of multimodal postoperative analgesia, it has a very strong research significance. However, in order to achieve the purpose of research, there are still some methodological problems. I have the following comments:

1. In the results, statistical methods should be presented in a way that meets the requirements of the journal. For example, whether to keep one decimal point or two decimal points, please unify the standard.
2. Repeat the question asked the first time. Tables should be presented in three lines.
