# Supplementary material for: Intraoperative intravenous low-dose esketamine improves quality of early recovery after laparoscopic radical resection of colorectal cancer: A prospective, randomized controlled trial
Source: PLoS One. 2023 Jun 2;18(6):e0286590. doi: 10.1371/journal.pone.0286590 (PMC10237502; doi:10.1371/journal.pone.0286590)
Supplement: Attachment — Submitted filename: Response to Reviewers.docx [file pone.0286590.s007.docx]

Detailed Response to Reviewers

Reference: PONE-D-22-33531

Title: Intraoperative intravenous low-dose esketamine improves quality of early recovery after laparoscopic radical resection of colorectal cancer: A prospective, randomized controlled trial

Journal title: PLOS ONE

Authors: Ying Xu, Long He, Shaoxuan Liu, Chaofan Zhang, Yanqiu Ai

Dear Editors:

Thank you for your useful comments on our manuscript. We wish to give a sincere gratitude to referees for reviewing our paper carefully. We apologize for any inconveniences caused by these errors. We have modified the manuscript accordingly, and the specific modifications are listed point by point below. The responses to the reviewers’ comments are marked using yellow highlight and presented following.

We would love to thank you for allowing us to resubmit a revised copy of the manuscript and we highly appreciate your time and consideration.

Best regards

Sincerely yours

Additional Editor Comments:

There are still major problem with the statistics, namely the sample size calculation. The authors changed the source of the data of their sample size calculation to a pilot study instead of a previous study. The authors need to clarify why they changed the source of the data. Do they think that the original calculation was not correct and they performed a post-hoc calculation?

Response: We are very grateful to your comments for the manuscript. Your suggestion are very important and have important guiding significance for my thesis writing and scientific research work. We are very sorry for your misunderstanding caused by the inappropriate expression we made in the manuscript. We didn’t change the source of the data of our sample size calculation. We calculated the sample size by combining the previous study and the preliminary findings. First, we determined the primary outcome based on the previous study, and then we determined the sample size based on the results of the pilot study.

**Thanks again for your advice and I hope to learn more from you.**

Reviewer: 1

Abstract

- Enumerate the adverse events in the method section of the abstract as you still mentioning it in the results and conclusion of the abstract for example “Other outcome include the incidence of postoperative adverse events (nausea, vomiting, dizziness, hallucination and pruritis)”

Response: Thank you for the suggestion. Your suggestion are very important and have important guiding significance for my thesis writing and scientific research work. The adverse events in the abstract has been removed.

**Thanks again for your advice and I hope to learn more from you.**

Introduction

- Please change colorectal patients to patients with colorectal cancer

Response: Thank you very much for the suggestion. Your suggestion are very important and have important guiding significance for my thesis writing and scientific research work. We have corrected it to patients with colorectal cancer. **Thanks again for your advice and I hope to learn more from you.**

Methods

- Change muscle relaxation to neuromuscular blockade.

Response: Thank you very much for the suggestion. Your suggestion are very important and have important guiding significance for my thesis writing and scientific research work. We have corrected it to neuromuscular blockade.

**Thanks again for your advice and I hope to learn more from you.**

The response to reviewer 3 point #2 is not clear

reviewer 3 point #2: So many p-values, and no adjustment for multiple testing. Yet you say 0.048 is significant. Without an adjustment for multiple testing, I don't believe your "significance" statements.

Response: Thank you very much for the suggestion. Your suggestion are very important and have important guiding significance for my thesis writing and scientific research work. Data at multiple time points, such as total QoR-15 scores, QoR-15 domains and VAS scores, were analyzed with an adjustment for multiple testing using Bonferroni or LSD, which was not specifically expressed in the statistical analysis part of the manuscript. Please see the figure below for specific statistical results on physical comfort.

**Thanks again for your advice and I hope to learn more from you.**

Reviewer: 3

1. In the results, statistical methods should be presented in a way that meets the requirements of the journal. For example, whether to keep one decimal point or two decimal points, please unify the standard.

Response: Thank you very much for the suggestion. Your suggestion are very important and have important guiding significance for my thesis writing and scientific research work. We have corrected it to unify the standard for keeping one decimal point.

**Thanks again for your advice and I hope to learn more from you.**

2. Repeat the question asked the first time. Tables should be presented in three lines.

Response: Thank you very much for the suggestion. Your suggestion are very important and have important guiding significance for my thesis writing and scientific research work. We have corrected the tables to be presented in three lines. And we are very sorry that the first answer was not satisfactory to you as we did not fully understand your question. Here are re-answer to the questions asked the first time.

**Thanks again for your advice and I hope to learn more from you.**

Point #1: You cite reference 18 as giving the sample size, but it's not clear to me that this trial had the same primary outcome, the same patient population, and the same variability that you had in your study. Can you justify using someone else's sample size computation? And if they already did the same study you did, why did you have to redo it?

Response: Thank you for the suggestion. We apologize for the inappropriate expression we made in the manuscript. Our study had the same primary outcome (the QoR-15 scores at 24 h after surgery) as reference 18, but the patient population and primary intervention are both different. We calculated the sample size by combining the previous study and the preliminary findings. First, we determined the primary outcome based on the reference 18, and then we determined the sample size based on the results of the pilot study.

**Thanks again for your advice and I hope to learn more from you.**

Point #2: So many p-values, and no adjustment for multiple testing. Yet you say 0.048 is significant. Without an adjustment for multiple testing, I don't believe your "significance" statements.

Response: Thank you very much for the suggestion. Data at multiple time points, such as total QoR-15 scores, QoR-15 domains and VAS scores, were analyzed with an adjustment for multiple testing using Bonferroni or LSD, which was not specifically expressed in the statistical analysis part of the manuscript. Please see the figure below for specific statistical results on physical comfort.

**Thanks again for your advice and I hope to learn more from you.**

Point #3: How do I know your sample size was adequate? It would be helpful to add to the discussion whether the assumptions underlying these computations were actually realized in the trial, but then you don't state the assumptions because they are taken from a different paper.

Response: Thank you for the suggestion. We used the PASS 15.0 to calculate the Power (1－β). Based on the mean, standard deviation (SD) and sample size of the two groups, the Power (1－β) calculated in PASS 15.0 was 0.99418. It is greater than 0.8. Therefore, our sample was adequate. Please see the figure below for specific calculation results.

**Thanks again for your advice and I hope to learn more from you.**

Point #4: Anytime you use repeated measures ANOVA, you need to give the assumptions of the model, and how you verified those assumptions (e.g., diagnostics).

Response: Thank you for the suggestion. In the statistical analysis section of this manuscript, we have shown that “*The Shapiro-Wilk test was used to test the data’s normality.* *Data with normal distribution at multiple time points were analyzed using repeated measures analysis of variance (ANOVA). Data with non-normal distribution at multiple time points were analyzed using the Generalized Estimating Equations (GEE)*”. And, the statistical analysis methods of many studies are also expressed in this way, such as the following literature: “Niu Zheng,Gao Xiuxiu,Shi Zeshu et al. Effect of total intravenous anesthesia or inhalation anesthesia on postoperative quality of recovery in patients undergoing total laparoscopic hysterectomy: A randomized controlled trial.[J] .J Clin Anesth, 2021, 73: 110374.” Specific statistical analysis is shown in the figure below.

**Thanks again for your advice and I hope to learn more from you.**

Reviewer #3: Many of my statistical comments were either not understood or not responded to adequately. The authors now admit that the paper that they based their sample size on is not relevant to this study, and now redo the sample size analysis from scratch. It seems odd to me that this new analysis gives the sample size used in the study. Typically, a study cannot be remediated when the design is incorrect. Secondly, the authors now conduct a post-hoc analysis using completely DIFFERENT techniques than were specified in the original analysis. The study was never powered for a correlation, it was powered by a difference in means. My comments on statistical significance and multiple testing was not addressed adequately. Distributional assumptions were not verified with diagnostics.

Response: We are very grateful to your comments for the manuscript. According with your advice, we amended the relevant part in manuscript. Your suggestion are very important and have important guiding significance for my thesis writing and scientific research work. We are very sorry that we did not adequately understand your statistical comments. We re-answer the questions asked the first time. We don’t redo the sample size analysis from scratch. We are very sorry for your misunderstanding caused by the inappropriate expression we made in the manuscript. We calculated the sample size by combining the previous study and the preliminary findings. First, we determined the primary outcome based on the previous study, and then we determined the sample size based on the results of the pilot study. Secondly, we used the PASS 15.0 to calculate the Power (1－β) to verify the adequacy of the sample size. Bonferroni was used as an adjustment for multiple testing.

We are very sorry that we did not fully understand the sentence “Distributional assumptions were not verified with diagnostics.” We tried to answer it in the Point #4 according to our understanding. Thanks again for your suggestion.

Thank you for your advice. It's very important. Due to your advice, I have found my shortcomings in my current work. I will improve my scientific research level and make more achievements in the future work according to your advice.

**Thanks again for your advice and I hope to learn more from you.**
