# Supplementary material for: Intraoperative intravenous low-dose esketamine improves quality of early recovery after laparoscopic radical resection of colorectal cancer: A prospective, randomized controlled trial
Source: PLoS One. 2023 Jun 2;18(6):e0286590. doi: 10.1371/journal.pone.0286590 (PMC10237502; doi:10.1371/journal.pone.0286590)
Supplement: Attachment — Submitted filename: Response to Reviewers.docx [file pone.0286590.s008.docx]

Detailed Response to Reviewers

Reference: PONE-D-22-33531

Title: Intraoperative intravenous low-dose esketamine improves quality of early recovery after laparoscopic radical resection of colorectal cancer: A prospective, randomized controlled trial

Journal title: PLOS ONE

Authors: Ying Xu, Long He, Shaoxuan Liu, Chaofan Zhang, Yanqiu Ai

Dear Editors:

Thank you for your useful comments on our manuscript. We wish to give a sincere gratitude to referees for reviewing our paper carefully. We apologize for any inconveniences caused by these errors. We have modified the manuscript accordingly, and the specific modifications are listed point by point below. The responses to the reviewers’ comments are marked using yellow highlight and presented following.

We would love to thank you for allowing us to resubmit a revised copy of the manuscript and we highly appreciate your time and consideration.

Best regards

Sincerely yours

Journal Requirements:

Please review your reference list to ensure that it is complete and correct. If you have cited papers that have been retracted, please include the rationale for doing so in the manuscript text, or remove these references and replace them with relevant current references. Any changes to the reference list should be mentioned in the rebuttal letter that accompanies your revised manuscript. If you need to cite a retracted article, indicate the article’s retracted status in the References list and also include a citation and full reference for the retraction notice.

Response: Thank you for the suggestion. In the reference list, the reference 5 was changed as suggested by reviewer 1 “In the first paragraph, you need to explain the operative related complication that could be improved by the study drug. Otherwise, it is better to remove the sentence starting with “However, the establishment…” as the role of the study drug is not to improve or impact those complications. You may consider other complications, such as inadequate pain management and use of opioids, which could be affected by the study drug.” during major revision. And, the reference 10 was added as suggested by reviewer 1 “In the second paragraph, mention some of the adverse effect of ketamine in comparison to the esketamine.” during major revision.

**Thanks again for your advice and I hope to learn more from you.**

Additional Editor Comments:

Thank you for your nice revision. There is a very minor statistical comment which needs correction before final acceptance of the manuscript. Well done.

Response: We are very grateful to your comments for the manuscript. We have made a correction in the statistical analysis section.

**Thanks again for your advice and I hope to learn more from you.**

Reviewer: 1

please state that "Adjustment for multiple testing was done using Bonferroni or LSD" in the statistical analysis section

Response: Thank you for the suggestion. Your suggestion are very important and have important guiding significance for my thesis writing and scientific research work. We have stated that “Adjustment for multiple testing was done using Bonferroni or LSD" in the statistical analysis section.

**Thanks again for your advice and I hope to learn more from you.**

Reviewer: 2

Thanks to the authors of the manuscript for their active and effective revisions. The quality of the manuscript has been greatly improved, basically meeting the requirements for publication. After my own careful examination, i feel that the native English needs to be further improved. If the language of the article is improved, I think the article is clinically significant and can be published.

Response: Thank you for the suggestion. I have consulted the teachers who is better at English around me to help make the revision, including some inaccuracies in the wording.

**Thanks again for your advice and I hope to learn more from you.**
